# Aspirated bile: a major host trigger modulating respiratory pathogen colonisation in cystic fibrosis patients

**DOI:** 10.1007/s10096-014-2133-8

**Published:** 2014-05-11

**Authors:** F. J. Reen, D. F. Woods, M. J. Mooij, M. N. Chróinín, D. Mullane, L. Zhou, J. Quille, D. Fitzpatrick, J. D. Glennon, G. P. McGlacken, C. Adams, F. O’Gara

**Affiliations:** 1 BIOMERIT Research Centre, School of Microbiology, University College Cork—National University of Ireland, Cork, Ireland; 2 Paediatric Cystic Fibrosis Clinic, Cork University Hospital, Cork, Ireland; 3 School of Chemistry and Analytical and Biological Chemistry Research Facility (ABCRF), University College Cork—National University of Ireland, Cork, Ireland; 4 School of Biomedical Sciences, Curtin University, Perth, WA 6845 Australia; 5 Present Address: Department of Medical Microbiology, Maastricht University Medical Centre, 6202 AZ Maastricht, The Netherlands

## Abstract

Chronic respiratory infections are a leading global cause of morbidity and mortality. However, the molecular triggers that cause respiratory pathogens to adopt persistent and often untreatable lifestyles during infection remain largely uncharacterised. Recently, bile aspiration caused by gastro-oesophageal reflux (GOR) has emerged as a significant complication associated with respiratory disease, and cystic fibrosis (CF) in particular. Based on our previous finding that the physiological concentrations of bile influence respiratory pathogens towards a chronic lifestyle in vitro, we investigated the impact of bile aspiration on the lung microbiome of respiratory patients. Sputum samples (*n* = 25) obtained from a cohort of paediatric CF patients were profiled for the presence of bile acids using high-resolution liquid chromatography–mass spectrometry (LC-MS). Pyrosequencing was performed on a set of ten DNA samples that were isolated from bile aspirating (*n* = 5) and non-bile aspirating (*n* = 5) patients. Both denaturing gradient gel electrophoresis (DGGE) and pyrosequencing revealed significantly reduced biodiversity and richness in the sputum samples from bile aspirating patients when compared with non-aspirating patients. Families and genera associated with the pervasive CF microbiome dominated aspirating patients, while bacteria associated with the healthy lung were most abundant in non-aspirating patients. Bile aspiration linked to GOR is emerging as a major host trigger of chronic bacterial infections. The markedly reduced biodiversity and increased colonisation by dominant proteobacterial CF-associated pathogens observed in the sputum of bile aspirating patients suggest that bile may play a major role in disease progression in CF and other respiratory diseases.

**Electronic supplementary material:**

The online version of this article (doi:10.1007/s10096-014-2133-8) contains supplementary material, which is available to authorized users.

## Introduction

Chronic respiratory infections associated with lung disease are a major cause of global morbidity and mortality (http://who.int/mediacentre/factsheets/fs310/en/). Despite concerted global research efforts, little progress has been made in the clinical management of these chronic infections. Current treatment strategies remain extremely limited, particularly where chronic pathogens adopt a biofilm lifestyle. Recent clinical investigations have highlighted bile aspiration into the lungs as a major consequence of gastro-oesopheageal reflux (GOR) [1–4]. GOR is routinely detected in respiratory patients, with incidences as high as 40–80 % reported for cystic fibrosis (CF) patients [4, 5]. GOR in CF patients is associated with reduced lung function manifesting as reduced forced expiratory volume (FEV) and forced vital capacity (FVC) [6, 7]. However, to date, the mechanism by which bile aspiration contributes to reduced pulmonary function remains unknown. Recently, we have shown that physiologically relevant concentrations of bile cause *Pseudomonas aeruginosa* and other respiratory pathogens to adopt a chronic biofilm lifestyle in vitro [8]. Furthermore, we have also demonstrated that bile acids modulate molecular targets in the host, suppressing the master regulator of the host hypoxic and immune response, HIF-1 (Legendre, C., Mooij, M., Adams, C. *et al*., manuscript in revision). This strongly suggests that bile aspiration into the lungs may be a major host determinant that triggers the establishment of dominant chronic biofilm-forming microbial species.

Several recent reports have highlighted the existence of a CF microbiome, with distinct microbial profiles from healthy non-CF patients [9–14]. While the factors that shape this CF microbiome remain unknown, our previous studies suggest that bile aspiration may influence the microbial diversity and, particularly, the emergence of dominant chronic pathogens in CF patients [8]. To investigate this, the bile aspiration status of patients was established using liquid chromatography–mass spectrometry (LC-MS) technology and the lung microbiome was subsequently investigated using 454 pyrosequencing. A cohort of paediatric CF patients was recruited for this study in order to gain insight into the early impact of bile aspiration on the emergence of chronic infection.

## Methods

### Patient cohort and ethics statement

Sputum samples (*n* = 25) were collected from paediatric patients attending the CF clinic at Cork University Hospital, Ireland, over a 6-month period (Table 1). Ethical approval was granted for sputum collection and all patients/guardians signed consent forms for acquisition and analysis outlined in this study. Sputum specimens were obtained by asking the patient to cough the specimen into a sterile universal container. Specimens were labelled with a unique study number. All sample collection was performed with the research group blinded to the patient data.

**Table 1:** Patient data for the paediatric patient cohort involved in the microbiome study

ID no.	Age (years)	Sex	IV antibiotics^a^	PO antibiotics^a^	FEV%^b^	Mutation	BA (μM)
1	17	M	28	56	90 %	ΔF508/ΔF508	0.34
2	15	F	28	14	85 %	ΔF508/ΔF508	0.50
3	8	M	0	56	104 %	ΔF508/ΔF508	0.50
4	11	M	30	CM + 56 days CP	85 %	ΔF508/ΔF508	0.16
5	12	M	14		97 %	ΔF508/ΔF508	0.28
6	16	M	21	CM + 28 days CP	86 %	ΔF508/ΔF508	0.1
7	16	M	14	CM	92 %	ΔF508/ΔF508	0.27
8	9	M	42	CM	89 %	ΔF508/G542	1.16
9	14	M	14	CM	92 %	ΔF508/ΔF508	0.53
10	16	F	70	*Ma* + PO CP	52 %	ΔF508/ΔF508	0.11
11	12	F	0	CM	79 %	ΔF508/G551D	0.55
12	9	M	65	CM. month on/off MT & AM	42 %	ΔF508/ΔF508	1.0
13	14	F	SC IV antibiotics	CM or CD	30 %	ΔF508/ΔF508	14.5
14	9	M	14	42 days	101 %	G55ID/Glu56Lys	1.22
15	7	F	42	CM	94 %	ΔF508/ΔF508	1.12
16	13	M	0	CM	75 %	ΔF508/G551D	0.04
17	13	M	36	CM + PO antibiotics	90 %	ΔF508/ΔF508	0.11
18^c^	11	M	14	CM	118 %	G55ID/ΔF508	1.0
19	7	F	14	CM	91 %	ΔF508/ΔF508	0.20
20	6	M	0	42 days	94 %	ΔF508^d^	0.18
21	19	M	28	100 days	84 %	ΔF508/ΔF508	GOR^e^ -ve
22	11	M	28	70 days	91 %	ΔF508/ΔF508	GOR -ve
23	15	M	28	0	96 %	ΔF508/G551D	GOR -ve
24	16	M	14	28 days	72 %	ΔF508/ΔF508	0.6
25	8	F	49	42 days	96 %	ΔF508/ΔF508	GOR +ve

CD, doxycycline; CM, chronic macrolide; PO, oral antibiotics; SC, semi-continuous

^a^Number of days in 2012

^b^Predicted baseline

^c^Patient commenced ivacaftor treatment

^d^Heterozygote, second mutation not identified

^e^Symptom-based classification

### Sample processing

Sample processing was performed using a method adapted from Tagliacozzi and colleagues [15]. Briefly, sputum samples were treated with equal volumes of Sputolysin (Calbiochem) and vortexed for 30 s prior to centrifugation at 5,000 rpm for 15 min. The supernatant was transferred into a sterile container and 250 μl was removed for bile acid analysis. A 900 μl aliquot of acetonitrile (Sigma-Aldrich) was added to each 250 μl sample, which was vortexed for 1 min and centrifuged at 13,600 rpm for 10 min. A 900 μl aliquot was transferred to a clean container and the sample was evaporated under nitrogen to dryness. The sample was then resuspended in 250 μl MeOH:H_2_O (1:1) and subsequently analysed by LC-MS.

### LC-MS bile acid analysis

Twelve bile acid standards were used and resuspended in methanol to a concentration of 20 mM; cholic acid (CA), chenodeoxycholic acid (CDCA), deoxycholic acid (DCA), lithocholic acid (LCA), ursodeoxycholic acid (UDCA), glycodeoxycholic acid (GDCA), taurochenodeoxycholic acid (TCDCA), taurodeoxycholic acid (TDCA), taurocholic acid (TCA), glycocholic acid (GCA) and taurolithocholic acid (TLCA) were purchased from Sigma-Aldrich (Buchs, Switzerland) and tauroursodeoxycholic acid (TUDCA) was purchased from Calbiochem (Darmstadt, Germany). The 12 stock solutions were then pooled together to a concentration of 100 μM in methanol. All chemicals used were HPLC grade.

Profiling of the bile composition in the clinical samples was undertaken using a recently developed LC-MS protocol (Reen, F.J., Woods, D.F., Zhou, L. *et al*., manuscript in preparation). Briefly, each sputum sample was analysed for the presence of 12 principal bile acids compared to purified standard. Patients were considered bile aspirating where concentrations of greater than 1.0 μM were detected. Baseline concentrations of less than 0.2 μM bile acids, comparable to healthy non-CF samples, were considered non-bile aspirating. Duplicate samples were included in the analysis to ensure reproducibility and all analyses were performed blinded to the patient data (Table 1).

### Denaturing gradient gel electrophoresis (DGGE) analysis

DNA extraction from the Sputolysin-treated sputum samples was achieved using the Puregene DNA isolation kit (Qiagen), according to the manufacturer’s instructions. DNA was RNase-treated to remove residual RNA and quantified to 100 ng/μl. Bacterial 16S rRNA amplification was performed using the universal primers 518R and F338GC, while the fungal internally transcribed region (ITS) was amplified using the ITS5F and ITS4R universal primers. The following polymerase chain reaction (PCR) programme was used for amplification: initial denaturation at 94 °C for 3 min, followed by 30 (bacterial) or 40 (fungal) cycles of 94 °C for 45 s, 52 °C for 45 s, and 72 °C for 1 min (bacterial) or 1.5 min (fungal). A final step of 72 °C for 6 min was included. Samples were loaded on a 10 % (bacterial) or 5 % (fungal) denaturing gel and cluster analysis of gel images was performed using Phoretix software (TotalLab, UK).

### 16S rRNA pyrosequencing analysis

DNA from Sputolysin-treated samples was quantified and standardised at a final concentration of 25 ng/μl. Samples were subsequently sent for 16S rRNA amplification and high-throughput sequencing using the Roche FLX Genome Sequencer in combination with Titanium Chemistry at DNAVision (Belgium). Detailed procedures are provided in Online Resource 1. Briefly, amplification of the V1–V3 region was achieved using the universal primers 518R 5′-ATTACCGCGGCTGCTGG-3′ and 27F 5′-AGAGTTTGATCCTGGCTCAG-3′ [16–18]. Amplicons were gel-purified and concentrations for all samples were determined by the PicoGreen assay. A nested PCR approach was taken in light of the low target DNA abundance of the samples, consistent with previous reports [19, 20]. Comparison of the biodiversity profiles from nested and single-step amplification on the same samples did not reveal any skew or distortion of the taxonomic distribution (data not shown). Between 5,476 and 18,290 raw sequence reads (Online Resource 2) were obtained from the nested amplification for each sputum sample (minimum read length of 430 bp), from which 3,435–11,671 passed QC analysis (Online Resource 3). Each sequence passing QC was assigned to a family by the Ribosomal Database Project (RDP) classifier (v 2.1) with confidence estimate (CE) > 80 % [21]. A minimum of 97 % was assigned at the phyla level, while more than 95 % of filtered 454 sequences was successfully classified down to the genus level. Richness and biodiversity indices based on operational taxonomic units (OTUs) were extracted using the mothur software platform [22]. The Chao1 index was used for richness estimation, related to the number of observed OTUs. Biodiversity related to how uniformly the sequences are spread into the different observed OTUs was estimated with the non-parametric Shannon formula. Both indices were evaluated at different distance unit cutoffs, to test different selectivities in the definition of OTUs.

### Statistical analysis

All data were analysed using Prism version 5.0 (GraphPad, San Diego, CA, USA) for statistical significance. GraphPad StatMate version 2.00 was used to establish that the study was adequately powered. As microbiome samples typically exhibit non-normal distributions, both a non-parametric Mann–Whitney *U*-test [95 % confidence interval (CI), one tailed] and an unpaired *t*-test with Welch correction were applied. Unless otherwise stated, the Welch corrected *t*-test is presented and indicates statistical relevance in both tests. A parametric Student’s *t*-test of unequal variance was performed on the DGGE data. In all cases, differences <0.05 were considered statistically significant.

## Results

### DGGE analysis of bacterial and fungal diversity in the paediatric CF cohort

**Fig. 1:**
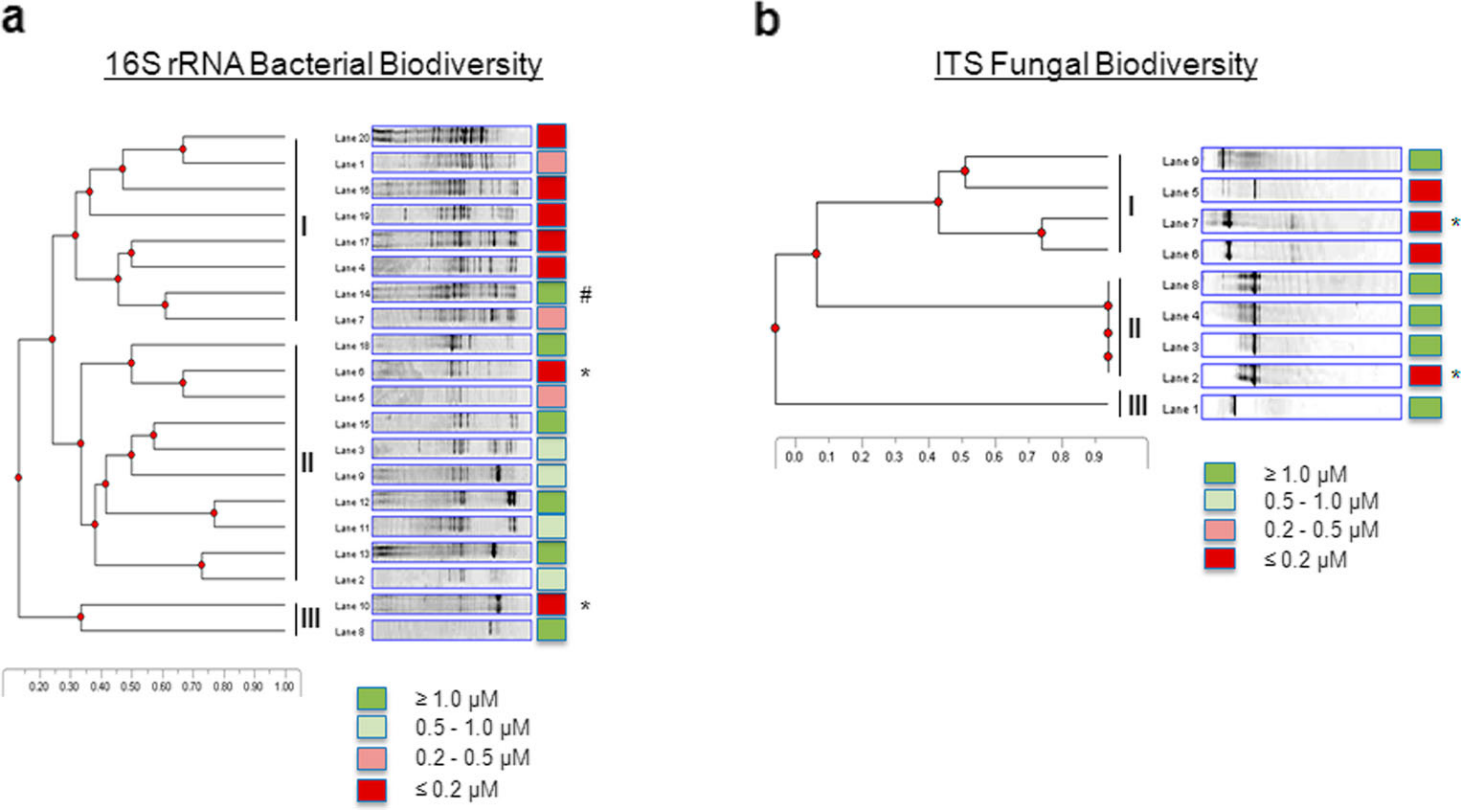
Denaturing gradient gel electrophoresis (DGGE) analysis of bacterial and fungal biodiversity in aspirating and non-aspirating paediatric cystic fibrosis (CF) patients. **a** Patient samples separated into three distinct clades. The lane numbers refer to the patient ID for comparison of bacterial profiles. Lane 14^#^ represents a patient that has only recently been diagnosed with CF and is an outlier to this analysis. Lanes 6* and 10* represent samples from patients that are symptomatic for gastro-oesophageal reflux disease (GORD). Statistical analysis was performed by Student’s *t*-test. **b** Fungal biodiversity profiles based on internally transcribed region (ITS) sequence variation. Lanes 7* and 2*, respectively, represent the GORD-positive outliers described above. The lane numbers refer to the following patient ID numbers in parentheses: Lane 1 (8), Lane 2 (10), Lane 3 (15), Lane 4 (13), Lane 5 (16), Lane 6 (19), Lane 7 (6), Lane 8 (12), Lane 9 (18)

To address the hypothesis that bile aspiration significantly influences the microbiology of the CF lung, DGGE profiling was performed on a paediatric patient cohort from Cork University Hospital, Ireland. Bacterial 16S rDNA profiles were initially analysed from sputum samples taken from nine patients and were correlated with the clinical classification of gastro-oesophageal reflux disease (GORD) status (Table 1 and Online Resource 4). A marked reduction in biodiversity was evident from those patients whose status was symptomatic for GORD compared to those who were asymptomatic. While this suggested that bile aspiration may, indeed, influence the biodiversity of the CF lung, there is a growing acceptance that bile aspiration may be asymptomatic and more accurate technologies are required to measure bile acids in the lungs. Therefore, in order to evaluate the occurrence and extent of bile aspiration in our paediatric CF cohort, a protocol for LC-MS bile acid analysis in sputum was developed (Reen, F.J., Woods, D., Zhou, L. *et al*., manuscript in preparation). Additional sputum samples were obtained from the paediatric CF cohort (*n* = 25), from which bacterial and fungal biodiversity profiles were generated. Patients were classified as either bile aspirating or non-aspirating on the basis of LC-MS analysis on sputum (Table 1). DGGE profiles clustered on the basis of aspiration status with marked reductions in bacterial biodiversity observed in aspirating samples relative to non-aspirating samples (Fig. 1a). Cluster analysis of fungal biodiversity profiles was less clear (Fig. 1b), although the paediatric samples were limited for fungal colonisation, which is unsurprising given the age profile of the cohort (Table 1). Taken together, this analysis suggested that bile aspiration into the lungs of paediatric CF patients had a significant influence on the microbiology of the lung.

### Pyrosequencing biodiversity analysis of the paediatric CF microbiome: aspirating vs. non-aspirating

**Fig. 2:**
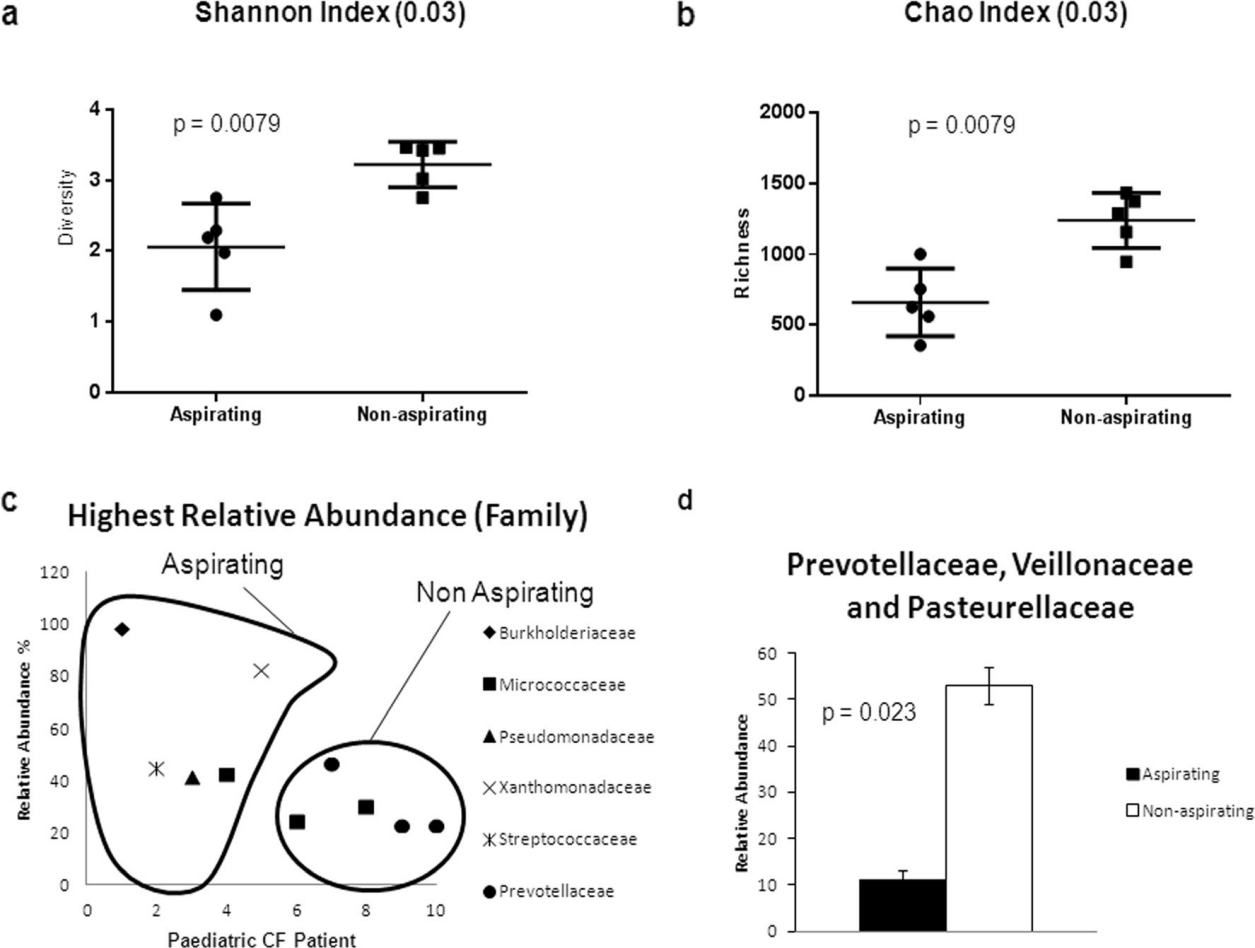
Microbiome analysis of bile aspirating and non-aspirating paediatric patients. **a** Diversity (non-parametric Shannon) and (**b**) richness (Chao1) indices from the pyrosequencing dataset. The figures are representative of five patient samples for both aspirating and non-aspirating [Mann–Whitney *U*-test (*p* < 0.05)]. **c** Family with the highest relative abundance in each patient sample. **d** Combined relative abundance of Prevotellaceae, Veillonellaceae and Pasteurellaceae in non-aspirating patients relative to aspirating patients [Student’s *t*-test (*p* < 0.05)]

In order to characterise the influence of bile aspiration on the CF microbiome, 454 pyrosequencing (DNAVision, Belgium) was performed (*n* = 10, Online Resource 5). On the basis of the sputum bile acid profiling, five bile aspirating and fi non-bile aspirating samples, matched for age and antibiotic profile, were selected. Indeed, analysis of the clinical and demographic data for bile aspirating and non-aspirating patients revealed no significant difference in FEV%, age or IV antibiotics between both cohorts (Online Resource 6). However, it should be noted that two of the lowest FEV% were recorded for aspirating patients, while one aspirating patient had commenced ivacaftor treatment (Table 1). The biodiversity index of each sample was calculated using the non-parametric Shannon algorithm and the bile aspirating samples were found to have a significantly lower diversity index (*p* = 0.0079) than the non-bile aspirating samples (Fig. 2a). This was further borne out by the Chao1 richness index, which was also statistically significantly reduced (Mann–Whitney *p* = 0.0079) in the bile aspirating samples (Fig. 2b). This reduction in the biodiversity index is comparable to previous reports from adult CF patients when compared with healthy non-CF patients [9, 11, 23]. In addition to the reduced biodiversity, another distinctive feature of the aspirating patient samples was the presence of dominant proteobacterial genera, while non-aspirating patients were characterised by the abundance of genera more associated with healthy non-CF lungs (Fig. 2c, d) [9].

### Phylum, family and genus level analysis: impact of bile aspiration

**Fig. 3:**
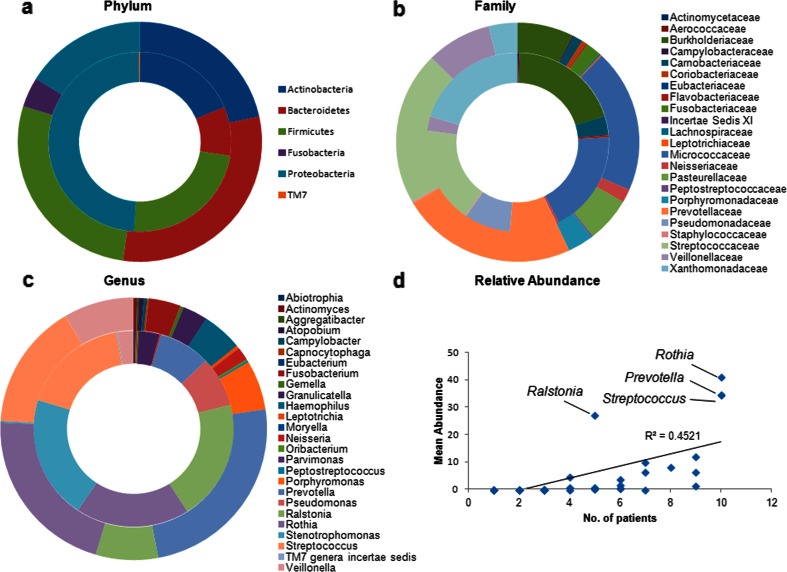
**a** Phylum, **b** family and **c** genus level analysis of the paediatric microbiome in aspirating and non-aspirating patients. In all cases, aspirating samples are represented by the *inner circle*, while non-aspirating samples are represented by the *outer circle*. Relative abundances ≥1 % are presented. **d** The relative abundance of genera was not linear relative to the patient number (*R* ^2^ = 0.4521), with *Ralstonia*, *Rothia*, *Streptococcus* and *Prevotella* being over-represented compared to other genera

The analysis of biodiversity at the phylum level revealed a significantly lower relative abundance of *Bacteroidetes* (*p* = 0.0135) and Fusobacteriaceae (*p* = 0.0291) in bile aspirating patients compared to non-aspirating, with the latter phylum being almost entirely absent (Fig. 3a and Online Resource 5). The ratio of *Firmicutes* to *Bacteroidetes*, previously shown to be a marker for the pervasive CF microbiome, was 5.13 (± 4.14) in aspirating patients and 1.11 (± 0.51) in non-aspirating patients. The analysis of family level diversity (Fig. 3b and Online Resource 5) revealed lower relative abundance of Veillonellaceae (*p* = 0.0173), Fusobacteriaceae (*p* = 0.04) and Prevotellaceae (*p* = 0.0422) in aspirating samples. Furthermore, Pasteurellaceae were significantly less prevalent in aspirating patients (*p* < 0.05).

Genus level analysis (Fig. 3c and Online Resource 5) revealed the prevalence of dominant proteobacterial species in 3/5 of the bile aspirating samples. These genera (*Ralstonia*, *Pseudomonas* and *Stenotrophomonas*) accounted for 45–98 % of the total microbiome in these patients. Additional bile aspirating patients were dominated by *Streptococcus* (45 %) and *Rothia* (43 %). In contrast, the non-bile aspirating patients contained significantly higher biodiversity, even where *Prevotella* was present at >40 %. Bile aspirating samples were notable for the almost exclusive absence (with the exception of patient 5, 0.1 % relative abundance) of *Haemophilus*, which was present in 4/5 non-aspirating samples. Furthermore, aspirating samples were also characterised by a lower relative abundance of *Veillonella* (*p* = 0.0123), *Prevotella* (*p* = 0.0462), *Fusobacteria* (*p* = 0.0391) and *Leptotrichia* (*p* = 0.0426) in these patients. Taken together, these data indicate a strong correlation between the aspiration of bile acids and the prevalence of CF-associated pathogens in the paediatric lung.

## Discussion

Several recent studies have investigated the lung microbiome in a broad spectrum of respiratory diseases. Despite differences in experimental design, e.g. sequencing technology and specimen type, there is strong concordance among the reports [9, 10, 20, 24–26]. Contrary to the perceived sterility of the healthy lung, microbial communities that may contribute to disease have been identified [9], while respiratory conditions such as CF, chronic obstructive pulmonary disease (COPD) and asthma were found to have distinct lung microbial profiles, both from each other and from the healthy lung [9, 10, 27]. A pattern emerging from these reports is the complexity of the microbial communities that exist in the different infection niches, leading to a re-evaluation of organisms that have traditionally been regarded as commensals. There is growing evidence that the diversity of these profiles may play a previously unforseen role in disease progression, in tandem with the well-studied pathogens such as *P. aeruginosa*, *H. influenzae* and *S. aureus* [9, 10, 13, 14].

Having established the profile of bile aspirating and non-aspirating patients within our CF cohort, the microbiome from these distinct patient groups was analysed. The suitability of sputum for this research has previously been established by several independent studies [9, 10, 28], which have shown that it reflects a microbiome distinct from the oropharyngeal tract and consistent with that of explanted lungs. Furthermore, recent bronchoalveolar lavage (BAL) data from Aseeri and colleagues confirms the presence of bile acids in the lower respiratory tract [1]. A key characteristic of the previously established CF microbiome is the markedly reduced biodiversity compared to non-CF samples, often correlating with the emergence of dominant pathogenic species within the lung. Our analysis shows that the bile aspirating patients exhibit markedly reduced biodiversity compared to non-aspirating patients, mirroring the outcome of the recent CF vs. non-CF microbiome studies (Fig. 2). Furthermore, in three of the aspirating samples, the reduced biodiversity correlated with the presence of a single dominant proteobacterial genus. This is particularly interesting and is consistent with our hypothesis that bile aspiration triggers the emergence of chronic-behaving dominant organisms that ultimately cause the chronic destruction of the lung [8].

Consistent with previous microbiome studies, the microbial profiles were quite diverse within the CF cohort [9]. Microbial families that were found to be more prevalent among CF patients, e.g. Pseudomonadaceae and Aerococcaceae [9, 13], were only present at relative abundances >1.0 % in bile aspirating patients. Conversely, Prevotellaceae, Veillonellaceae, Fusobacteriaceae and Leptotrichiaceae, which are more prevalent in healthy non-CF patients [9, 13], were significantly more abundant in non-aspirating patients. The apparent suppression of genera that require anaerobic conditions for growth, such as *Veillonella* and *Prevotella*, is highly significant, given that previous studies have shown that almost half of the cultured CF airway microbial community in adult sputum is composed of obligate anaerobes [13]. Furthermore, several studies have shown that the administration of antibiotic regimens targeting aerobic pathogens does not appear to significantly alter the anaerobic microflora in CF patients [29, 30]. It is interesting, therefore, to speculate that the reduction of anaerobic bacteria in aspirating patients may arise as a consequence of the emergence of dominant proteobacterial pathogens, particularly in light of our recent finding that bile causes respiratory pathogens to adopt a chronic lifestyle [8]. Alternatively, anaerobic bacteria may be more sensitive to bile acids, and this will need to be followed up by future in vitro studies.

The possibility that antibiotic treatment is responsible for the lower biodiversity in aspirating patients was considered but is not supported by the evidence. The antibiotic regimens of all ten patients included in this study were comparable, although the non-aspirating samples underwent, on average, a shorter period of IV antibiotic treatment (Table 1 and Online Resource 6). Furthermore, given that most CF patients undergo prolonged antibiotic treatments, the administration of additional antibiotics during exacerbation are not likely to cause significant changes in the microbial community structure [9]. Also, importantly, the consistency of the replicates included in the study, which were taken at least 2 months apart (Online Resource 4 and Fig. 1), strongly supports the hypothesis that bile aspiration is a major influence on microbial biodiversity in the CF lung.

This study provides strong evidence that bile aspiration is a major factor in shaping the CF microbiome, from an early stage in paediatric patients. While consistent with the influence of bile on the chronic behaviour of respiratory pathogens [8], further studies will be required on a larger patient cohort to establish whether the association between bile aspiration and reduced microbial biodiversity is causative or correlative. Further analysis of co-morbidities associated with GORD, such as gastrointestinal disease and chronic cough, will be undertaken, while the integration of BAL samples into the analysis will provide additional confirmation of the response in the lower respiratory tract. It is important to note that, while the current study focused on CF patients, the problems associated with bile aspiration are far more widespread in respiratory disease. The aspiration of bile acids has been confirmed in sputum and BAL samples from patients suffering from a range of other respiratory diseases, including COPD and asthma, and following lung transplantation [31, 32]. Studies have also found a significant correlation between GOR and *P. aeruginosa* infection [33, 34], as well as reduced lung function in CF and other respiratory patients following lung transplantation [35, 36]. Current treatment strategies for the management of GOR may not be effective in preventing the subsequent influence on the respiratory microbiome. The most effective solution for the prevention of GOR and bile aspiration is surgery, specifically Nissen fundoplication, although the high risks associated with this approach highlight the need for alternatives. Therefore, alternative innovative therapeutic strategies will be required in order to target the link between this key host trigger and the onset of chronic infection. This highlights the need for a large-scale study of bile aspiration in paediatric patients with CF and other respiratory conditions, with the specific aim of characterising the influence of bile aspiration on the microbiology of the lung and elucidating the molecular mechanisms underpinning changes in the microbial community structure.

## Electronic supplementary material

Below are the links to the electronic supplementary material.

ESM 1 (PDF 132 kb)

ESM 2 (PDF 126 kb)

ESM 3 (PDF 123 kb)

ESM 4 DGGE analysis of bacterial 16S rDNA profiles from paediatric sputum samples. 16S rDNA amplicons from nine paediatric sputum samples from individual patients were analysed on a denaturing gradient gel. GORD status was based on clinical observation and patient data. A marked reduction in biodiversity was evident in patients that were categorised as GORD symptomatic relative to those that were asymptomatic. Amplicons A and B were identified as *Rothia* and *Pseudomonas* genera, respectively (PDF 40 kb)

ESM 5 Microbiome biodiversity analysis of phyla, family and genera from sputum samples of individual paediatric CF patients. Each section of the bar chart represents the relative abundance in the individual sputum sample. Relative abundances ≥1% are presented (PDF 217 kb)

ESM 6 (PDF 14 kb)
