# Supplementary material for: Aspirated bile: a major host trigger modulating respiratory pathogen colonisation in cystic fibrosis patients
Source: Eur J Clin Microbiol Infect Dis. 2014 May 11;33(10):1763–71. doi: 10.1007/s10096-014-2133-8 (PMC4182646; doi:10.1007/s10096-014-2133-8)
Supplement: Supplementary file 1 — (PDF 132 kb) [file 10096_2014_2133_MOESM1_ESM.pdf]

European Journal of Clinical Microbiology and Infectious Diseases: Original Article

Aspirated bile: a major host trigger modulating respiratory pathogen colonisation in Cystic Fibrosis patients.

F. Jerry Reen[1], David F. Woods[1], Marlies J. Mooij[1¥], Muireann Ní Chróinín[2], David Mullane[2], Lin Zhou[3], Jonathan Quille[3], Dara Fitzpatrick [3], Jeremy D. Glennon[3], Gerard P. McGlacken[3], Claire Adams[1] and Fergal O'Gara[1,4*].

[1] BIOMERIT Research Centre, School of Microbiology, University College Cork - National University of Ireland, Cork, Ireland.

[2] Paediatric Cystic Fibrosis Clinic, Cork University Hospital, Cork, Ireland.

[3] School of Chemistry and Analytical and Biological Chemistry Research Facility (ABCRF), University College Cork - National University of Ireland, Cork, Ireland.

[4] Curtin University, School of Biomedical Sciences, Perth WA 6845, Australia.

[¥] Present address: Maastricht University Medical Centre, Department of Medical Microbiology, AZ Maastricht, The Netherlands.

Running Title: Bile aspiration modulates biodiversity.

***** To whom correspondence should be addressed. Mailing address: Prof. Fergal O'Gara, BIOMERIT Research Centre, School of Microbiology, University College Cork, Ireland. Phone number: + 353-21-4901315; Fax number: + 353-21-4275934; E. mail: f.ogara@ucc.ie.

DNA Quality Control.

DNA quality was controlled through O.D. 260/280 ratio by nanodrop (Nanodrop Inc.) and the DNA was quantified using the Quant-iT PicoGreen dsDNA reagent and kit (Life Tech, Carlsbad, USA), following the manufacturer's instructions.

rRNA pyrosequencing.

16S rRNA genes were amplified at DNAVision using a primer set corresponding to primers ATTACCGCGGCTGCTGG and AGAGTTTGATCCTGGCTCAG. These PCR primers target the V1-V3 hypervariable 16S rRNA region. The forward primer contained the sequence of the Titanium A adaptor (5′-CCATCTCATCCCTGCGTGTCTCCGACTCAG-3′) and a barcode sequence (pools of 8 samples). For each sample, a PCR mix of 100 µl was prepared containing 1 × PCR buffer, 2U of KAPA HiFi Hotstart polymerase blend and dNTPs (Kapabiosystems), 300 nM primers (Eurogentec, Liege, Belgium), and 60 ng gDNA. Thermal cycling consisted of initial denaturation at 95°C for 5 min, followed by 25 cycles of denaturation at 98°C for 20 s, annealing at 56°C for 40 s, and extension at 72°C for 20 s, with a final extension of 5 min at 72°C. Aliquots (3 µl) of PCR product were added to a new PCR mix (identical to the first round of PCR) for the nested PCR of 15 cycles. Amplicons were visualised on 1% agarose gels using GelGreen Nucleic Acid gel stain in 1x TAE (Biotium) and were cleaned using the Wizard SV Gel and PCR Clean-up System (Promega), according to the manufacturer's instructions.

Amplicon Quantitation, Pooling, and Pyrosequencing.

Amplicon DNA concentrations were determined using the Quant-iT PicoGreen dsDNA reagent and kit (Life Tech, Carlsbad, USA) following the manufacturer's instructions. Assays were carried out using 2 µl cleaned PCR product in a total reaction volume of 200 µl in black,

96-well microtiter plates. Following quantitation, cleaned amplicons were combined in equimolar ratios into a single tube. The final pool of DNA was eluted in 100 µl of nuclease free water, was purified using an Agencourt Ampure XP Purification system (Agencourt Biosciences Corporation-Beckman coulter, USA) and then resuspended in 100 µl of TE 1x. The concentration of the purified pooled DNA was determined using the Quant-iT PicoGreen dsDNA reagent and kit (Life Tech, Carlsbad, USA). Pyrosequencing was carried out using primer A on a 454 Life Sciences Genome Sequencer FLX instrument (Roche) following titanium chemistry.

16S rRNA data analysis.

The sequences were assigned to samples according to sample-specific barcodes. This enabled collection of FASTA formatted files containing an average (± SD) of 10,597 ± 3625 sequences per sample. Sequences were then checked for the following criteria: (i) almost perfect match with barcode and primers; (ii) length of at least 240 nucleotides (barcodes and primers excluded); (iii) no more than two undetermined bases (denoted by N). The term "almost perfect match" signifies that one mismatch/deletion/insertion is allowed in the barcode, idem for the primer. Each sequence originating from pyrosequencing and passing QC was assigned to a family by the Ribosomal Database Project (RDP) classifier (v 2.1) with CE > 80%. The Shannon diversity and Chao richness estimates were calculated through the Mothur package.
