# Supplementary material for: Aspirated bile: a major host trigger modulating respiratory pathogen colonisation in cystic fibrosis patients
Source: Eur J Clin Microbiol Infect Dis. 2014 May 11;33(10):1763–71. doi: 10.1007/s10096-014-2133-8 (PMC4182646; doi:10.1007/s10096-014-2133-8)
Supplement: Supplementary file 2 — (PDF 126 kb) [file 10096_2014_2133_MOESM2_ESM.pdf]

European Journal of Clinical Microbiology and Infectious Diseases: Original Article

Aspirated bile: a major host trigger modulating respiratory pathogen colonisation in Cystic Fibrosis patients.

F. Jerry Reen[1], David F. Woods[1], Marlies J. Mooij[1¥], Muireann Ní Chróinín[2], David Mullane[2], Lin Zhou[3], Jonathan Quille[3], Dara Fitzpatrick [3], Jeremy D. Glennon[3], Gerard P. McGlacken[3], Claire Adams[1] and Fergal O'Gara[1,4*].

[1] BIOMERIT Research Centre, School of Microbiology, University College Cork - National University of Ireland, Cork, Ireland.

[2] Paediatric Cystic Fibrosis Clinic, Cork University Hospital, Cork, Ireland.

[3] School of Chemistry and Analytical and Biological Chemistry Research Facility (ABCRF), University College Cork - National University of Ireland, Cork, Ireland.

[4] Curtin University, School of Biomedical Sciences, Perth WA 6845, Australia.

[¥] Present address: Maastricht University Medical Centre, Department of Medical Microbiology, AZ Maastricht, The Netherlands.

Running Title: Bile aspiration modulates biodiversity.

***** To whom correspondence should be addressed. Mailing address: Prof. Fergal O'Gara, BIOMERIT Research Centre, School of Microbiology, University College Cork, Ireland. Phone number: + 353-21-4901315; Fax number: + 353-21-4275934; E. mail: f.ogara@ucc.ie.

Table ESM1. 454 GS-FLX Sequencing Read Statistics.

**Patient ID**	**nReads**	**nBases**	**lMed**	**lMax**	**lMin**	**lAvg**	**sAvg**
13	5476	2252202	511	555	30	411.29	33.24
12	11337	4968405	500	815	32	438.25	33.55
18	9690	4172740	494	554	30	430.62	32.41
15	7274	3160616	492	538	31	434.51	33.11
8	8849	4091829	511	556	30	462.41	32.75
4	8012	3471190	490	610	30	433.25	33.01
16	13153	6103908	508	722	34	464.07	33.16
20	18290	7487322	490	973	35	409.37	33.34
19	12478	5359394	496	674	30	429.51	33.19
17	11409	4836457	497	660	30	423.92	32.99

nReads: number of reads; nBases: number of bases; lMed: median read length; lMax: maximum read length; lMin: minimum read length; lAvg: average read length; sAvg: average quality score (Phred score).
