# Supplementary material for: Aspirated bile: a major host trigger modulating respiratory pathogen colonisation in cystic fibrosis patients
Source: Eur J Clin Microbiol Infect Dis. 2014 May 11;33(10):1763–71. doi: 10.1007/s10096-014-2133-8 (PMC4182646; doi:10.1007/s10096-014-2133-8)
Supplement: Supplementary file 3 — (PDF 123 kb) [file 10096_2014_2133_MOESM3_ESM.pdf]

European Journal of Clinical Microbiology and Infectious Diseases: Original Article

Aspirated bile: a major host trigger modulating respiratory pathogen colonisation in Cystic Fibrosis patients.

F. Jerry Reen[1], David F. Woods[1], Marlies J. Mooij[1¥], Muireann Ní Chróinín[2], David Mullane[2], Lin Zhou[3], Jonathan Quille[3], Dara Fitzpatrick [3], Jeremy D. Glennon[3], Gerard P. McGlacken[3], Claire Adams[1] and Fergal O'Gara[1,4*].

[1] BIOMERIT Research Centre, School of Microbiology, University College Cork - National University of Ireland, Cork, Ireland.

[2] Paediatric Cystic Fibrosis Clinic, Cork University Hospital, Cork, Ireland.

[3] School of Chemistry and Analytical and Biological Chemistry Research Facility (ABCRF), University College Cork - National University of Ireland, Cork, Ireland.

[4] Curtin University, School of Biomedical Sciences, Perth WA 6845, Australia.

[¥] Present address: Maastricht University Medical Centre, Department of Medical Microbiology, AZ Maastricht, The Netherlands.

Running Title: Bile aspiration modulates biodiversity.

***** To whom correspondence should be addressed. Mailing address: Prof. Fergal O'Gara, BIOMERIT Research Centre, School of Microbiology, University College Cork, Ireland. Phone number: + 353-21-4901315; Fax number: + 353-21-4275934; E. mail: f.ogara@ucc.ie.

Table ESM2: Reads Quality Information.

Patient ID	nReads	lMed	lMax	lMin	lAvg
13	3435	487	537	430	483
12	8289	487	538	430	479
18	6546	482	535	430	476
15	5285	473	516	430	476
8	6928	488	538	430	484
4	5768	474	519	430	473
16	10253	489	525	430	481
20	11671	487	544	430	479
19	8763	481	536	430	476
17	7710	488	524	430	480
