# Supplementary material for: Aspirated bile: a major host trigger modulating respiratory pathogen colonisation in cystic fibrosis patients
Source: Eur J Clin Microbiol Infect Dis. 2014 May 11;33(10):1763–71. doi: 10.1007/s10096-014-2133-8 (PMC4182646; doi:10.1007/s10096-014-2133-8)
Supplement: Supplementary file 4 — DGGE analysis of bacterial 16S rDNA profiles from paediatric sputum samples. 16S rDNA amplicons from nine paediatric sputum samples from individual patients were analysed on a denaturing gradient gel. GORD status was based on clinical observation and patient data. A marked reduction in biodiversity was evident in patients that were categorised as GORD symptomatic relative to those that were asymptomatic. Amplicons A and B were identified as Rothia and Pseudomonas genera, respectively (PDF 40 kb) [file 10096_2014_2133_MOESM4_ESM.pdf]

European Journal of Clinical Microbiology and Infectious Diseases: Original Article

Aspirated bile: a major host trigger modulating respiratory pathogen colonisation in Cystic Fibrosis patients.

F. Jerry Reen[1], David F. Woods[1], Marlies J. Mooij[1¥], Muireann Ní Chróinín[2], David Mullane[2], Lin Zhou[3], Jonathan Quille[3], Dara Fitzpatrick [3], Jeremy D. Glennon[3], Gerard P. McGlacken[3], Claire Adams[1] and Fergal O'Gara[1,4*].

[1] BIOMERIT Research Centre, School of Microbiology, University College Cork - National University of Ireland, Cork, Ireland.
[2] Paediatric Cystic Fibrosis Clinic, Cork University Hospital, Cork, Ireland.
[3] School of Chemistry and Analytical and Biological Chemistry Research Facility (ABCRF), University College Cork - National University of Ireland, Cork, Ireland.
[4] Curtin University, School of Biomedical Sciences, Perth WA 6845, Australia.

[¥] Present address: Maastricht University Medical Centre, Department of Medical Microbiology, AZ Maastricht, The Netherlands.

Running Title: Bile aspiration modulates biodiversity.

***** To whom correspondence should be addressed. Mailing address: Prof. Fergal O'Gara, BIOMERIT Research Centre, School of Microbiology, University College Cork, Ireland. Phone number: + 353-21-4901315; Fax number: + 353-21-4275934; E. mail: f.ogara@ucc.ie.

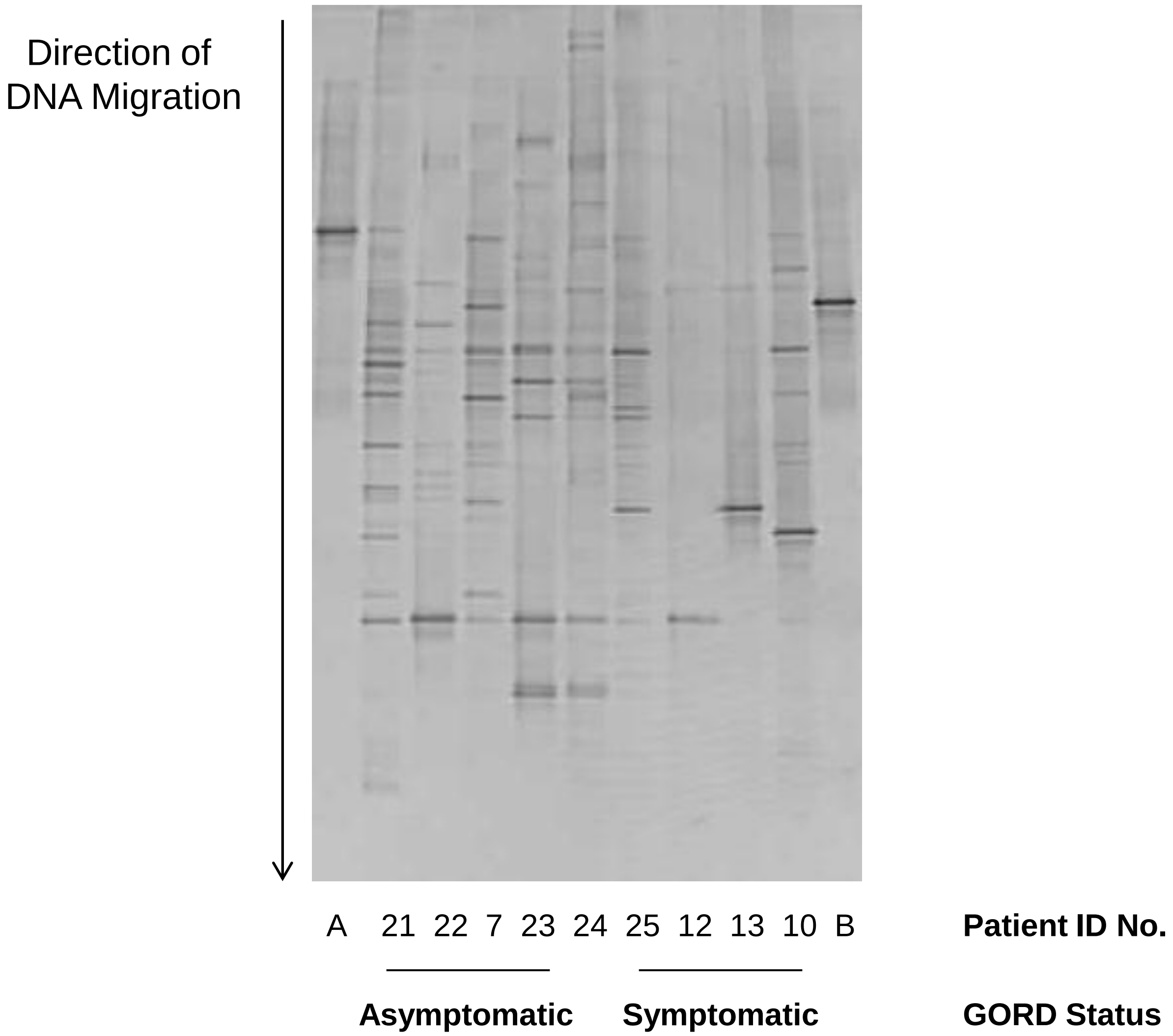

ESM3 DGGE analysis of bacterial 16S rDNA profiles from paediatric sputum samples 16S rDNA amplicons from 9 paediatric sputum samples from individual patients were analysed on a denaturing gradient gel. GORD status was based on clinical observation and patient data. A marked reduction in biodiversity was evident in patients that were categorised as GORD symptomatic relative to those that were asymptomatic. Amplicons A and B were identified as *Rothia* and *Pseudomonas* genera, respectively
