# Supplementary material for: Aspirated bile: a major host trigger modulating respiratory pathogen colonisation in cystic fibrosis patients
Source: Eur J Clin Microbiol Infect Dis. 2014 May 11;33(10):1763–71. doi: 10.1007/s10096-014-2133-8 (PMC4182646; doi:10.1007/s10096-014-2133-8)
Supplement: Supplementary file 5 — Microbiome biodiversity analysis of phyla, family and genera from sputum samples of individual paediatric CF patients. Each section of the bar chart represents the relative abundance in the individual sputum sample. Relative abundances ≥1% are presented (PDF 217 kb) [file 10096_2014_2133_MOESM5_ESM.pdf]

European Journal of Clinical Microbiology and Infectious Diseases: Original Article

Aspirated bile: a major host trigger modulating respiratory pathogen colonisation in Cystic Fibrosis patients.

F. Jerry Reen[1], David F. Woods[1], Marlies J. Mooij[1¥], Muireann Ní Chróinín[2], David Mullane[2], Lin Zhou[3], Jonathan Quille[3], Dara Fitzpatrick[3], Jeremy D. Glennon[3], Gerard P. McGlacken[3], Claire Adams[1] and Fergal O'Gara[1,4*].

[1] BIOMERIT Research Centre, School of Microbiology, University College Cork - National University of Ireland, Cork, Ireland.
[2] Paediatric Cystic Fibrosis Clinic, Cork University Hospital, Cork, Ireland.
[3] School of Chemistry and Analytical and Biological Chemistry Research Facility (ABCRF), University College Cork - National University of Ireland, Cork, Ireland.
[4] Curtin University, School of Biomedical Sciences, Perth WA 6845, Australia.

[¥] Present address: Maastricht University Medical Centre, Department of Medical Microbiology, AZ Maastricht, The Netherlands.

Running Title: Bile aspiration modulates biodiversity.

***** To whom correspondence should be addressed. Mailing address: Prof. Fergal O'Gara, BIOMERIT Research Centre, School of Microbiology, University College Cork, Ireland. Phone number: + 353-21-4901315; Fax number: + 353-21-4275934; E. mail: f.ogara@ucc.ie.

Phylum

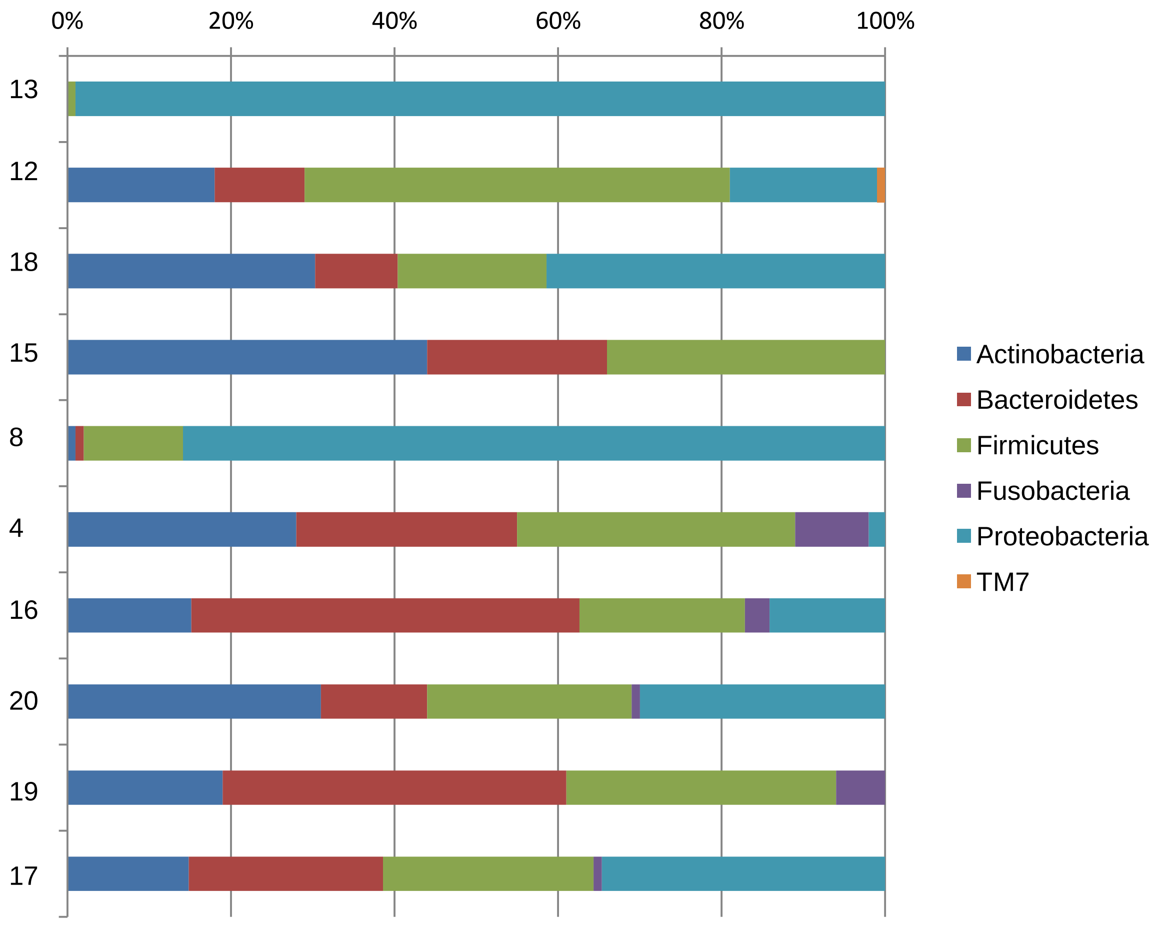
0%
20%
40%
60%
80%
100%
13
12
18
15
8
4
16
20
19
17
Actinobacteria
Bacteroidetes
Firmicutes
Fusobacteria
Proteobacteria
TM7

Family

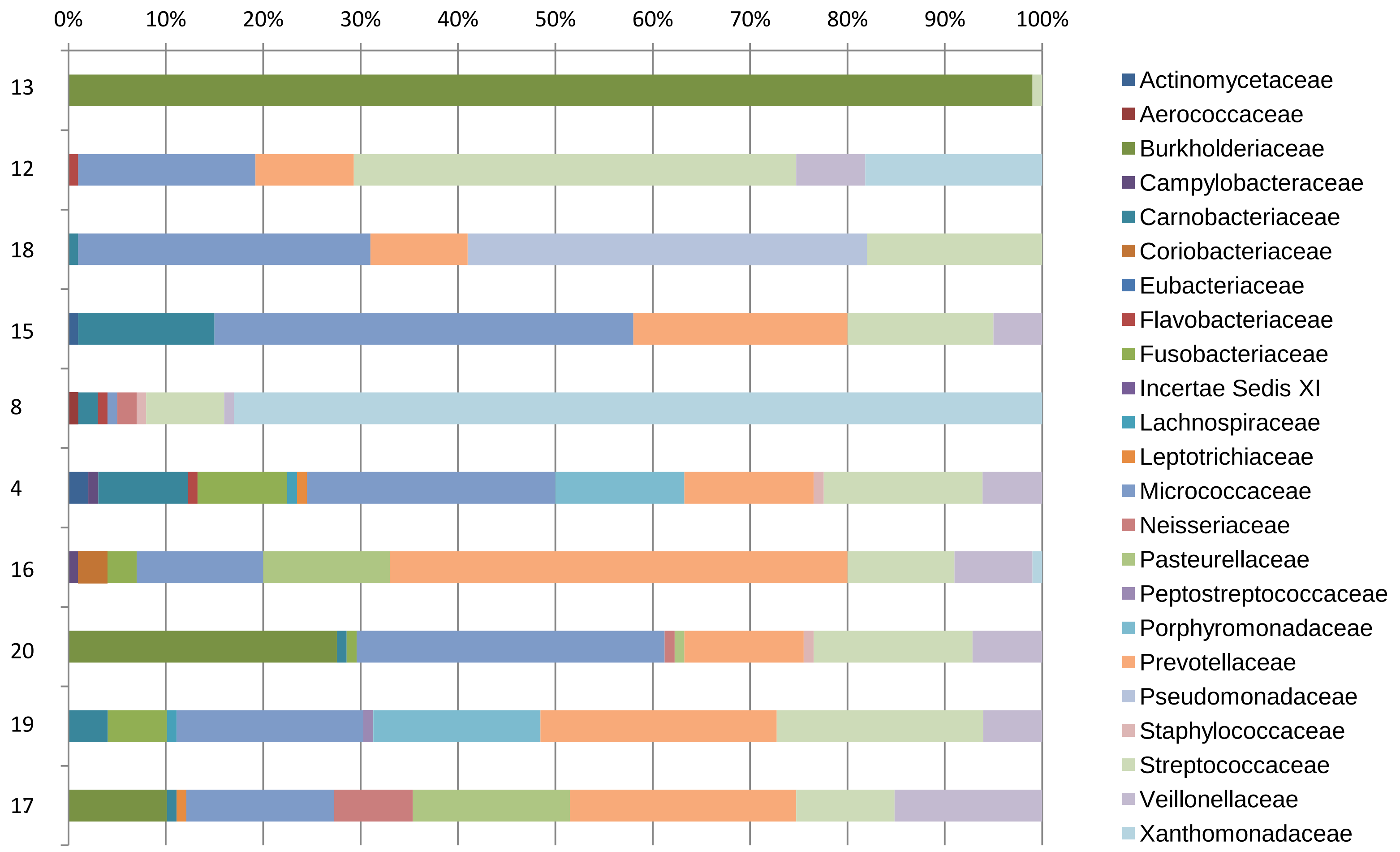

0%
10%
20%
30%
40%
50%
60%
70%
80%
90%
100%
13
12
18
15
8
4
16
20
19
17
Actinomycetaceae
Aerococcaceae
Burkholderiaceae
Campylobacteraceae
Carnobacteriaceae
Coriobacteriaceae
Eubacteriaceae
Flavobacteriaceae
Fusobacteriaceae
Incertae Sedis XI
Lachnospiraceae
Leptotrichiaceae
Micrococcaceae
Neisseriaceae
Pasteurellaceae
Peptostreptococcaceae
Porphyromonadaceae
Prevotellaceae
Pseudomonadaceae
Staphylococcaceae
Streptococcaceae
Veillonellaceae
Xanthomonadaceae

Genus

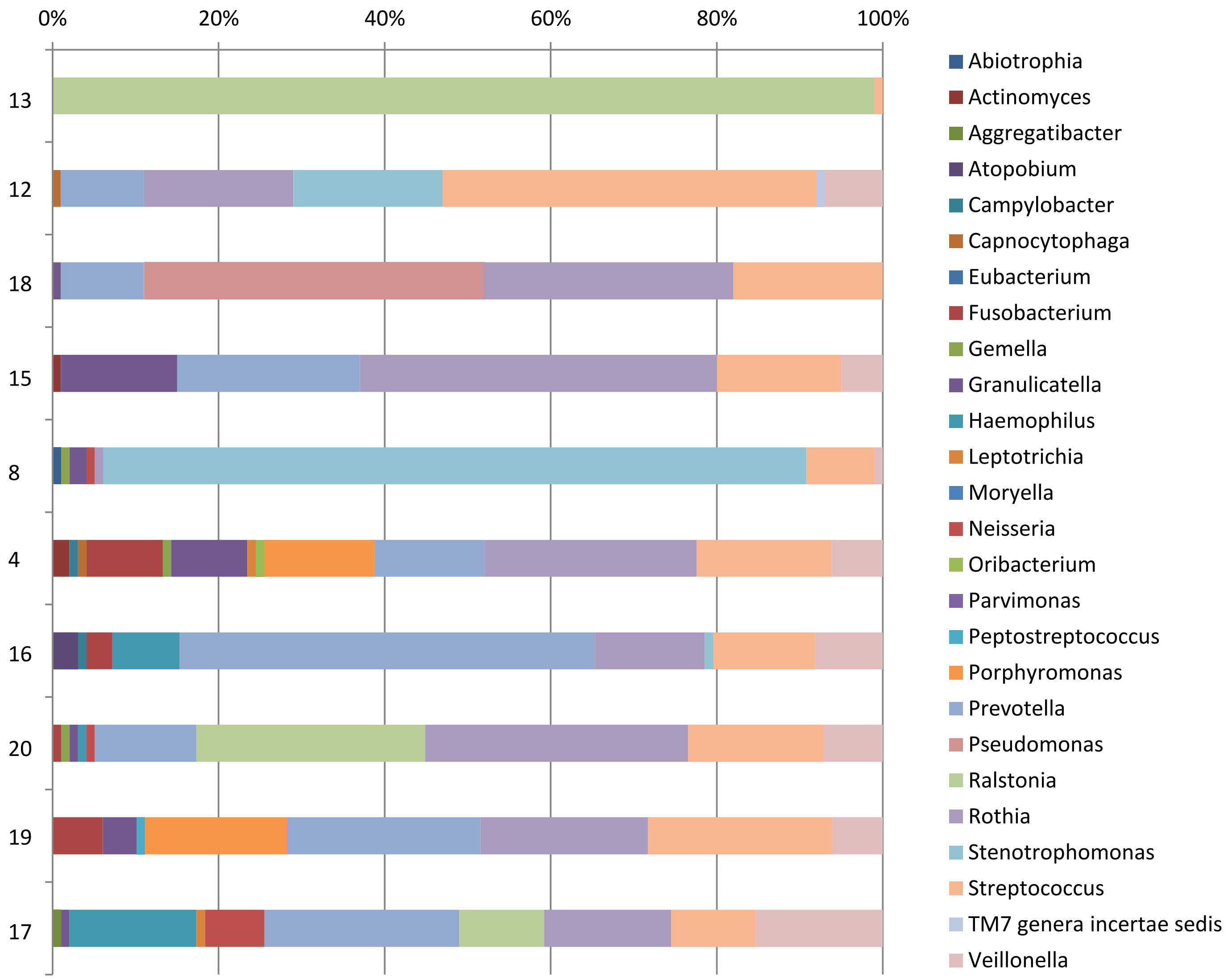

0%
20%
40%
60%
80%
100%
13
12
18
15
8
4
16
20
19
17
Abiotrophia
Actinomyces
Aggregatibacter
Atopobium
Campylobacter
Capnocytophaga
Eubacterium
Fusobacterium
Gemella
Granulicatella
Haemophilus
Leptotrichia
Moryella
Neisseria
Oribacterium
Parvimonas
Peptostreptococcus
Porphyromonas
Prevotella
Pseudomonas
Ralstonia
Rothia
Stenotrophomonas
Streptococcus
TM7 genera incertae sedis
Veillonella

ESM4 Microbiome biodiversity analysis of Phyla, Family and Genera from sputum samples of individual paediatric CF patients. Each section of the bar chart represents the relative abundance in the individual sputum sample
