# Supplementary material for: Aspirated bile: a major host trigger modulating respiratory pathogen colonisation in cystic fibrosis patients
Source: Eur J Clin Microbiol Infect Dis. 2014 May 11;33(10):1763–71. doi: 10.1007/s10096-014-2133-8 (PMC4182646; doi:10.1007/s10096-014-2133-8)
Supplement: Supplementary file 6 — (PDF 14 kb) [file 10096_2014_2133_MOESM6_ESM.pdf]

ESM 6. Clinical and demographic analysis of bile-profiled patients.

<0.5 µM	**Mean**	**StdDev**
n=10		
Male n=8		
Female n=2		
Age	12.7	3.831159
Mean FEV	85%	0.131217
Mean IV antibiotics	22.7	20.42901

>0.5 µM	**Mean**	**StdDev**
n=11		
Male n=7		
Female n=4		
Age	11.27273	3.101319
Mean FEV	82%	0.261812
Mean IV antibiotics	23.3	20.79557

<0.2 µM	**Mean**	**StdDev**
n=7		
Male n=5		
Female n=2		
Mean Age	11.71429	3.988077
Mean FEV	82%	0.145078
Mean IV antibiotics	24.42857	24.34377

>1.0 µM	**Mean**	**StdDev**
n=6		
Male n=4		
Female n=2		
Mean Age	9.833333	2.401388
Mean FEV	79%	0.349285
Mean IV antibiotics	35.4	21.67487
